# The new normal: Medical education during and beyond the COVID-19 pandemic

**DOI:** 10.36834/cmej.70317

**Published:** 2020-12-07

**Authors:** Rahim Kachra, Allison Brown

**Affiliations:** 1 Cumming School of Medicine, University of Calgary, Alberta, Canada

The “new normal” is a trite axiom that has permeated every sphere impacted by the COVID-19 pandemic, within and beyond healthcare. Healthcare has quickly adopted necessary changes in the delivery of care, using virtual clinical encounters, recruiting an expanded workforce, disrupting longstanding hospital processes, and embracing the palpable existential angst associated with this “new normal.”

Medical learners have also been significantly impacted at this time. The experiences of medical students and residents across the globe generally vary based on clinical, regional, and personnel needs. Medical schools and residency programs have had to quickly respond and adapt to the spread of the pandemic by making rapid decisions with the best interests of faculty, staff, learners, and the public in mind. End-users, such as learners, appear to have been excluded from these decision-making processes. In response to the pandemic, medical schools and residency programs have removed medical students from the clinical environment, re-distributed residents throughout the health system, and moved learning and assessments to online platforms. This represents a substantial disruption to the status quo of medical education.

Other major emergencies that have disrupted medical education include the severe acute respiratory syndrome (SARS) pandemic in 2003 and natural disasters such as Hurricane Katrina in 2005. One study reported that medical student performance declined following the restructuring of the curriculum in response to Hurricane Katrina.^1^ The literature about the impact of global emergencies or disasters on medical students is somewhat limited, yet, it is important to consider how learners might be impacted at this time. First, nearly all teaching and learning has become virtual, including the efficient adoption of virtual technologies to deliver content to learners remotely or asynchronously, and the implementation of online assessment modalities.^2^ Whether or not these current approaches are comparable remains unclear, but these strategies may be the only feasible options that can simultaneously support the continuation of teaching and maintain physical distancing. Second, clinical clerkships have also been considerably modified. At many institutions, the vast majority of clerks have been removed from clinical rotations; at others, final year clerks are being asked to graduate early and begin residency immediately so they can help provide patient care. For some final year residents, the postponement of their licensing examinations has impacted their career trajectories and has potentially influenced their employment.

The pandemic has also impacted the mental health and wellbeing of the public, and we anticipate similar challenges with mental health in both healthcare and medical education. Reports have already shed light on the mental health burden on healthcare workers during COVID-19.^3^ Medical learners are particularly prone to burnout and may be at an increased risk for mental health issues, such as depression and suicidal ideation.^4^ In addition to these mental health concerns, many medical learners are being asked to work in environments that may not have adequate supplies of personal protective equipment, which poses a physical safety risk to learners and patients. Already, one medical learner in the United States has died as a result of COVID-19. Medical schools must prioritize the physical safety risks to their learners with patient safety in parallel with considerations for the potential impact of these events on learners.

COVID-19 is not our first pandemic, nor will it likely be our last. However, the global impact should inspire the medical education community not only to identify opportunities for medical education, but also to create actionable plans that can be embedded into international curricula. Our research team is currently conducting an international, cross-sectional survey of medical students and residents to broadly examine the impact of the COVID-19 pandemic on medical learners. While our study remains ongoing concurrent with the pandemic, we wish to highlight the ways that medical education has been impacted thus far and articulate several early recommendations for medical educators to consider at the moment as well as for future events, such as a dreaded second wave. We offer the following considerations:

1. Prior to this seemingly forced transition to virtual medical education, there was a global re-imagination of the content, structure, and delivery of medical education. The pandemic led to rapid responses and changes to the delivery of medical training, including the uptake of virtual technologies that had long been resisted, and even criticized. We now have a basis to ground this work in, and we can explore novel modalities to provide ongoing medical education. These should be embedded as a part of curricula beyond the pandemic, so that they can persist through the next interruption to medical education.
2. Involve undergraduate and postgraduate medical learners in this process, including in decision-making, and work with them to best utilize their skill sets. Medical students are typically considered a homogenous group of learners; however, many come from different educational backgrounds (e.g., nursing, military, public health). Many may be ready and willing to volunteer.^5^ Upon first analysis of our study, it is clear that many medical learners feel under-utilized and want to help out at this time, and some schools already have structured processes in place to allow medical students to volunteer with initiatives such as contact tracing. We should capitalize on these individual strengths and motivations. Medical students at the University of Toronto during SARS in 2003 wanted to remain engaged in care and were proud to contribute during such stressful circumstances.^6^ It is important to consider that these experiences during the COVID-19 pandemic could potentially better equip our current learners to be leaders in various fields, such as public health.
3. Recognize the potential mental health implications and respond proactively. A variety of emotions are already associated with this pandemic: ‘Pre-Traumatic Stress Disorder’ (PreTSD), existential angst, fear and concern for our colleagues, patients, and families, dealing with the death of colleagues, and managing the duty to serve with your own limitations, among others. Trainees also feel this impact, and it is in our power to support them. Medical schools and residency programs should consistently and persistently reach out to their learners during this time instead of waiting for learners to reach out to them.
4. Communicate early, clearly, and often. Learners are anxious about the evolving impact on not only their learning and progression, but also their potential utilization to provide care as the pandemic spreads. This recommendation is consistent with a lesson learned from the SARS pandemic in 2003.^6^ We suggest that institutions continue to provide frequent and transparent communications with their learners, including acknowledging when they might not have a clear answer or response to learners.

The COVID-19 pandemic has substantially impacted medical education as we knew it. It is vital that we collaborate with, and learn from, each other. We encourage the global medical education community to continue to communicate with one another, share what is working, and spread innovative ideas so we can not only be successful in this ‘new normal,’ but learn from this experience to improve education moving forward.
